# Efficacy of mesenchymal stromal cells in the treatment of type 1 diabetes: a systematic review

**DOI:** 10.1007/s10561-024-10128-1

**Published:** 2024-02-21

**Authors:** Jiaxin Liu, Yang Yang, Yun Qi

**Affiliations:** Department of Endocrinology, Tianyou Hospital, Wuhan University of Science and Technology, Wuhan, 430034 China

**Keywords:** Type 1 diabetes, Stem cell, Cell transplantation, Systematic review

## Abstract

To investigate the efficacy of mesenchymal stromal cells in the treatment of type 1 diabetes. Articles about the effects of mesenchymal stromal cells for T1D were retrieved in PubMed, Web of Science, Embase, and the Cochrane Library databases up to July 2023. Additional relevant studies were manually searched through citations. HbA1c, FBG, PBG, insulin requirement and C-peptide were assessed. The risk of bias was evaluated with the ROB 2.0 and ROBINS-I tools. Six RCTs and eight nRCTs were included. Of the 14 studies included, two evaluated BM-MSCs, three evaluated UC-MSCs, five evaluated AHSCT, two evaluated CB-SCs, and two evaluated UC-SCs plus aBM-MNCs. At the end of follow-up, ten studies found that mesenchymal stromal cells improved glycemic outcomes in T1D, while the remaining four studies showed no significant improvement. Findings support the positive impacts observed from utilizing mesenchymal stromal cells in individuals with T1D. However, the overall methodological quality of the identified studies and findings is heterogeneous, limiting the interpretation of the therapeutic benefits of mesenchymal stromal cells in T1D. Methodically rigorous research is needed to further increase credibility.

**Supplementary Information:**

The online version contains supplementary material available at 10.1007/s10561-024-10128-1.

## Introduction

Type 1 diabetes (T1D) is an autoimmune metabolic disorder that occurs when beta cells in the pancreas are destroyed, resulting in insulin deficiency, hyperglycaemia, and clinical symptoms including polyphagia, polydipsia, polyuria as well as weight loss (Atkinson et al. 2014). The global incidence of T1D has been steadily increasing with a prevalence rate of 9.5% (Mobasseri et al. 2020). It is well-known that individuals with T1D face long-term complications, higher mortality rates, and shorter lifespan than that of the general population (Goodkin 1975). Despite the availability of exogenous insulin therapy, achieving optimal glycemic control remains challenging in T1D patients at risk for chronic complications. This places a significant burden on both individuals with T1D and their families (Powers 2021). While pancreas or islet transplantation can effectively manage various diabetes-related complications through metabolic control, challenges such as donor scarcity and graft rejection response need to be addressed before widespread application (Gremizzi et al. 2010; Matsumoto 2010). Previous studies have demonstrated the ability of human embryonic stem cells (hESCs) as well as induced pluripotent stem cells (iPSCs) to generate pancreatic progenitors or functional beta cells (León-Quinto et al. 2004; Pellegrini et al. 2016; Soria et al. 2000). In a recent clinical trial assessing the efficacy of pancreatic endoderm cells (PECs) in patients with T1D under immune suppression, Viacyte’s PEC-Direct combination product was subcutaneously administrated, managing to establish a beta cell mass and improve glucose control in T1D patients (Keymeulen et al. 2023). Specifically, some research has shown that mesenchymal stromal cells (MSCs) reduce glycosylated hemoglobin levels (HbA1c), decrease reliance on exogenous insulin dosages and increase C-peptide levels (Cai et al. 2016; Hu et al. 2013). However, there have been trials where no improvement in those reported outcomes was discovered in individuals with T1D (Ghodsi et al. 2012; Giannopoulou et al. 2014; Lu et al. 2021). Therefore, our systematic review aims at evaluating the benefit of various MSCs in treating T1D patients, focusing on laboratory parameters like HbA1c, fasting blood glucose (FBG), postprandial blood glucose (PBG), insulin requirements, and C-peptide.

## Methods

Our systematic review was reported under the Preferred Reporting Items for Systematic Reviews and Meta-analyses (PRISMA) statement guidelines (Moher et al. 2009), registered with the International Prospective Register of Systematic Reviews (PROSPERO): CRD42023453688.

### Search strategy

Articles about the efficacy of MSCs on T1D were retrieved in four electronic databases (Web of Science, PubMed, Embase, and the Cochrane Library) up to July 2023. Medical Subject Heading (MeSH) terms were combined with keywords to search for potentially relevant studies, including "type 1 diabetes mellitus" and "stem cell", without any restrictions. In addition, a reference list of the identified papers was manually screened in order to identify other relevant publications. The detailed search strategy is provided in Online Resource 1.

### Eligibility criteria

The Population, Intervention, Comparison, Outcomes and Study design (PICOS) framework contributed to establish the inclusion criteria: (1) Population: participants diagnosed with T1D; (2) Intervention: MSCs; (3) Comparison: routine therapy or placebo; (4) Outcomes: HbA1c, C-peptide, FBG, PBG and insulin requirements; (5) Study design: randomized controlled trials (RCTs) as well as non-randomized parallel controlled trials.

These papers were excluded if they involved: (1) other types of diabetes; (2) MSCs in combination with other unconventional treatment; (3) in-vitro studies and animal experiments; (4) case reports, reviews, letters, editorials, commentaries, theses, dissertations, study protocols and conference abstracts; (5) unpublished research.

### Study selection

Endnote X9 software was utilized to import the retrieved citations. Two authors independently removed duplicates and reviewed the headings and abstracts. Following a preliminary screening, full texts of the documents were thoroughly reviewed and selected on the basis of eligibility criteria. If necessary, we attempted to solve any disagreements through discussion with the third author.

### Data extraction

Data from each paper were separately extracted and collected by two authors, containing the following content: the primary author, year of publication, registered country, study design, diabetes duration, the number of participants, gender, age, body mass index (BMI), intervention details (type, dose, injection method) follow‐up period and results. Divergence was discussed with a third reviewer, when required.

### Quality assessment

Two independent reviewers evaluated the methodological quality of each study, using the Cochrane Collaboration tool. RCTs were determined by means of the Cochrane Risk of Bias 2.0 (RoB 2.0) tool (Sterne et al. 2019), which focused on the randomization process, deviations from intended interventions, missing outcome data, measurement of the outcome, and selection of the reported result. To assess the bias of each trial, "low risk", "some concerns", or "high risk" was assigned to each item. For non-randomized controlled trials (nRCTs), the Risk of Bias In Non-randomized Studies-of Interventions (ROBINS-I) tool was applied (Sterne et al. 2016). Biases due to confounding, participant selection, measurement of interventions, deviation from intended interventions, missing data, evaluation of outcomes, and selective reporting of outcomes were evaluated. Each "signaling question" was categorized into "low", "moderate", "serious", "critical", or "no information". Discrepancies were settled through discussion, and a third reviewer was involved if necessary.

## Results

### Search results

**Fig. 1:**
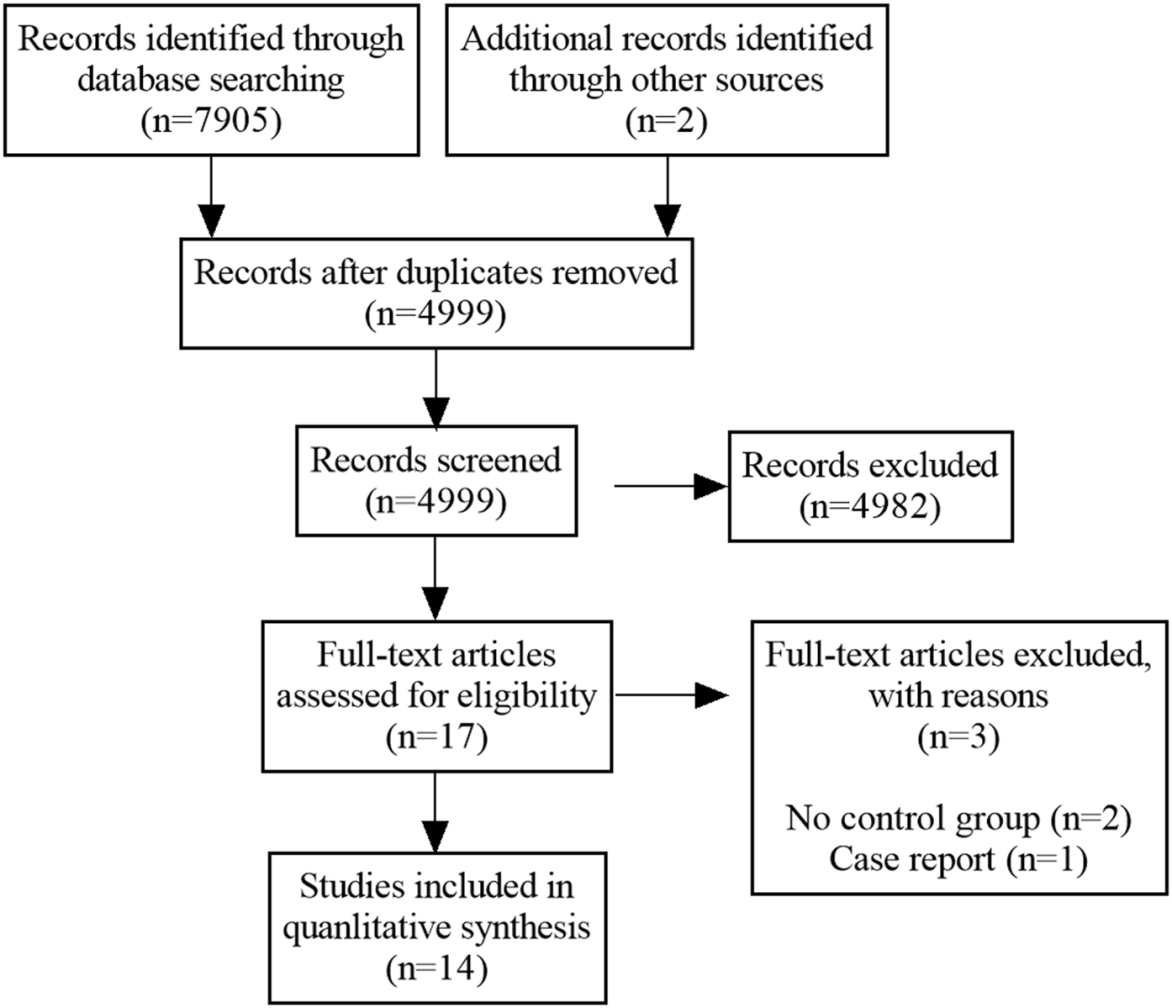
PRISMA flowchart of study selection process

The initial search identified a total of 7907 articles, of which 7905 were from the electronic database and 2 were from the citation search. Elimination of duplicates resulted in 4999 headings and abstracts. 17 studies were then selected for a comprehensive evaluation of the full text. Of these, three articles in accordance with the exclusion criteria were excluded, which either was a case report (n = 1), or lacked a control group (n = 2). Finally, 14 eligible trials were enrolled in the systematic review (Cai et al. 2016; Carlsson et al. 2015; Ghodsi et al. 2012; Giannopoulou et al. 2014; Gu et al. 2014, 2017; Hu et al. 2013; Izadi et al. 2022; Lu et al. 2021; Walicka et al. 2018; Wu et al. 2022; Ye et al. 2017; Yu et al. 2011; Zhao et al. 2012). The PRISMA flowchart of study selection process is illustrated in Fig. 1.

### Study characteristics

All 14 studies were published between 2011 and 2022. The trials took place in various countries, including Sweden, Iran, China, Poland and Germany. All studies were designed in parallel. Of the 14 articles included, six were RCTs and eight were nRCTs. Of 397 included subjects, 202 participated in MSCs intervention, and 195 were in control. One study did not mention the gender of the participants, and in the remaining 13 studies, the proportion of males ranged from 35.0 percent to 72.2 percent. Mean baseline BMI was reported in 12 studies, ranging from 15.63 to 23.3 kg/m^2^. The population had a mean or median age of 3 to 35 years, a history of diabetes between less than 3 months and 16.5 years, and a follow-up of 9 months to 8 years. Two articles used bone marrow-derived mesenchymal stromal cells (BM-MSCs) as an intervention, three used umbilical cord-derived mesenchymal stromal cells (UC-MSCs), five used autologous hematopoietic stem cell transplantation (AHSCT), one used umbilical cord blood (UCB), one used cord blood-derived stem cells (CB-SCs), and two used UC-MSCs plus autologous bone marrow-derived mononuclear cells (aBM-MNCs). Most cells were administered via peripheral intravenous injection, whereas two RCTs used dorsal pancreatic arteries. To analyze outcomes, 11 studies compared the MSCs group to a control group, while two studies compared to baseline. Table 1 demonstrates the characteristics of these trials.

**Table 1:** Characteristics of included studies

Author and Year	Country	Study Design	Group	Simple size (Male/female)	Age (years)	BMI (kg/m^2^)	Diabetes duration	Injection method	Mean dose of injection (cells/kg)	Follow-up period (months)
Carlsson et al. (2015)	Sweden	RCT	BM-MSCs	9 (8/1)	24 ± 6	23.3 ± 3.3	< 3 weeks	IV	2.1–3.6 × 10^6^	12
			Control	9 (5/4)	27 ± 6	22.5 ± 2.7				
Izadi et al. (2022)	Iran	RCT	BM-MSCs	11 (6/5)	10.27 ± 1.67	16.75 ± 2.57	Newly diagnosed	IV	1 × 10^6^	12
			Control	10 (5/5)	11.50 ± 2.63	18.91 ± 3.41				
Hu et al. (2013)	China	RCT	UC-MSCs	15 (9/6)	17.6 ± 33.7	20.9 ± 14.3	Not more than 6 months	IV	2.6 ± 1.2 × 10^7^	24
			Control	14 (8/6)	18.2 ± 29.6	21.3 ± 15.7				
Lu et al. (2021)	China	nRCT	UC-MSCs	27 (12/15)	22.4 ± 13.9	18.7 ± 3.6	2.3 (0.9, 7.7) months	IV	1.0 × 10^6^	12
			Control	26 (13/13)	27.4 ± 14.3	19.1 ± 3.5	1.0 (0.1, 7.6) months			
Yu et al. (2011)	China	nRCT	UC-MSCs	6 (3/3)	19.67 ± 2.58	21.56 ± 1.67	Less than 3 months	IV	1 × 10^7^/person	9
			Control	6 (4/2)	14.83 ± 8.18	20.08 ± 3.48				
Ghodsi et al. (2012)	Iran	RCT	AHSCT	15 (9/6)	21.61 ± 10.53	NA	(4.23 ± 2.21) years	IV	7–11 × 10^6^	12
			Control	15 (6/9)	21.35 ± 9.80		(4.11 ± 2.86) years			
Gu et al. (2014)	China	nRCT	AHSCT	14 (5/9)	8.04 ± 3.99	16.31 ± 3.25	Newly diagnosed	IV	NA	60
			Control	28 (10/18)	8.29 ± 2.91	15.63 ± 1.66				
Gu et al. (2017)	China	nRCT	AHSCT	20 (7/13)	18 ± 3.9	18.5 ± 1.45	(68 ± 45.5) days	IV	NA	48
			Control	20 (7/13)	18 ± 4.5	18.8 ± 2.29	(51 ± 37.8) days			
Walicka et al. (2018)	Poland	nRCT	AHSCT	23 (NA)	25 ± 5	22.5 ± 2.3	Newly diagnosed	IV	> 3.0 × 10^6^	48
			Control	8 (NA)	26 ± 3	23.0 ± 5.1				
Ye et al. (2017)	China	nRCT	AHSCT	8 (3/5)	18.86 ± 1.46	19.25 ± 1.11	< 6 months	IV	NA	12
			Control	10 (4/6)	20.18 ± 4.02	18.28 ± 1.39				
Giannopoulou et al. (2014)	Germany	nRCT	UCB	7 (5/2)	3.02 (1.82–5.38)	NA	101 (73–348) days	IV	1.27 (0.43–2.27) × 10^6^	12
			Control	10 (4/6)	6.60 (3.59–10.85)		139 (63–410) days			
Zhao et al. (2012)	China	nRCT	CB-SCs	12 (3/9)	28.17 ± 9.34	23.2 ± 3.4	(8.50 ± 6.36) years	IV	NA	10
			Control	3 (3/0)	33 ± 9	22.0 ± 2.7	(6 ± 7) years			
Cai et al. (2016)	China	RCT	UC-MSCs + aBM-MNCs	21 (9/12)	18.29 ± 5.22	21.99 ± 1.78	(9.24 ± 4.75) years	IP	1.1 × 10^6^ UC-MSCs plus 106.8 × 10^6^ aBM-MNCs	12
			Control	21 (11/10)	20.38 ± 3.67	22.06 ± 2.46	(7.00 ± 3.26) years			
Wu et al. (2022)	China	RCT	UC-MSCs + aBM-MNCs	14 (6/8)	34.2 ± 5.2	21.9 ± 1.8	(16.5 ± 4.7) years	IP	1.10 ± 0.22 × 10^6^ UC-MSCs plus 0.61 ± 0.26 × 10^10^ aBM-MNCs	96
			Control	15 (9/6)	35.5 ± 5.8	22.3 ± 2.46	(15.4 ± 3.4) years			

### Risk of bias assessment

**Fig. 2:**
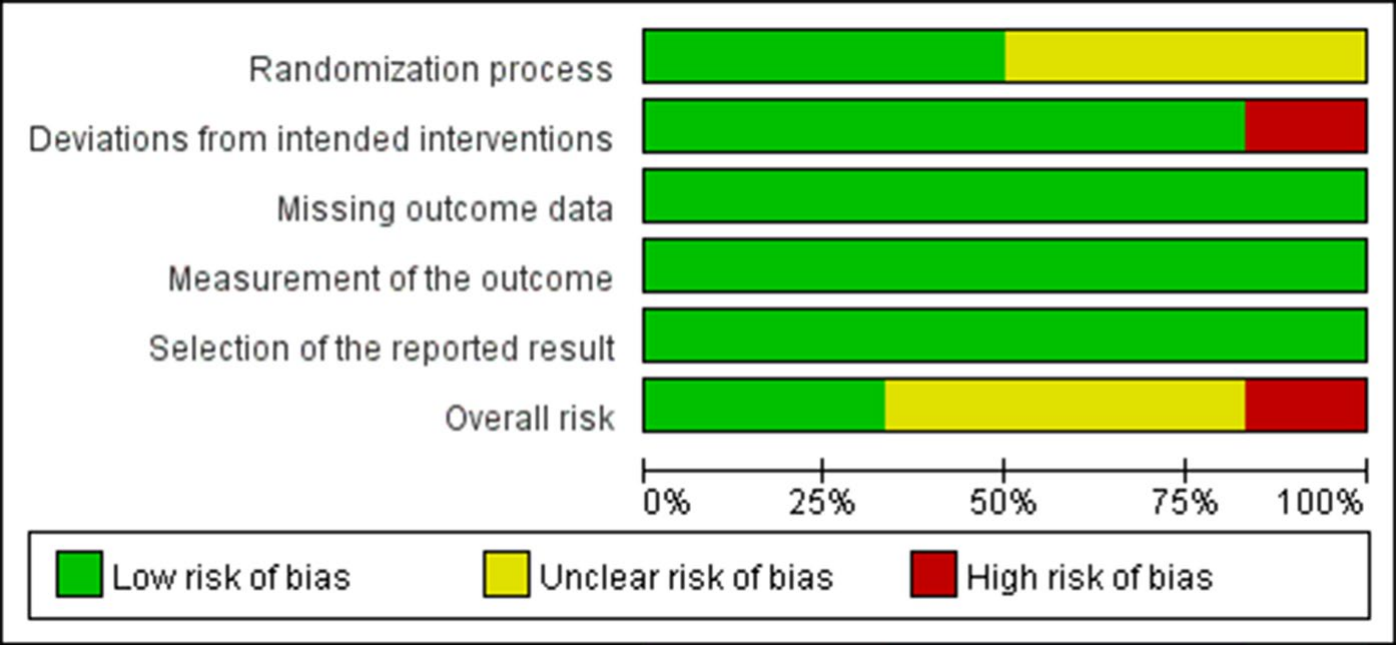
Risk of bias graph for randomized controlled trials

**Fig. 3:**
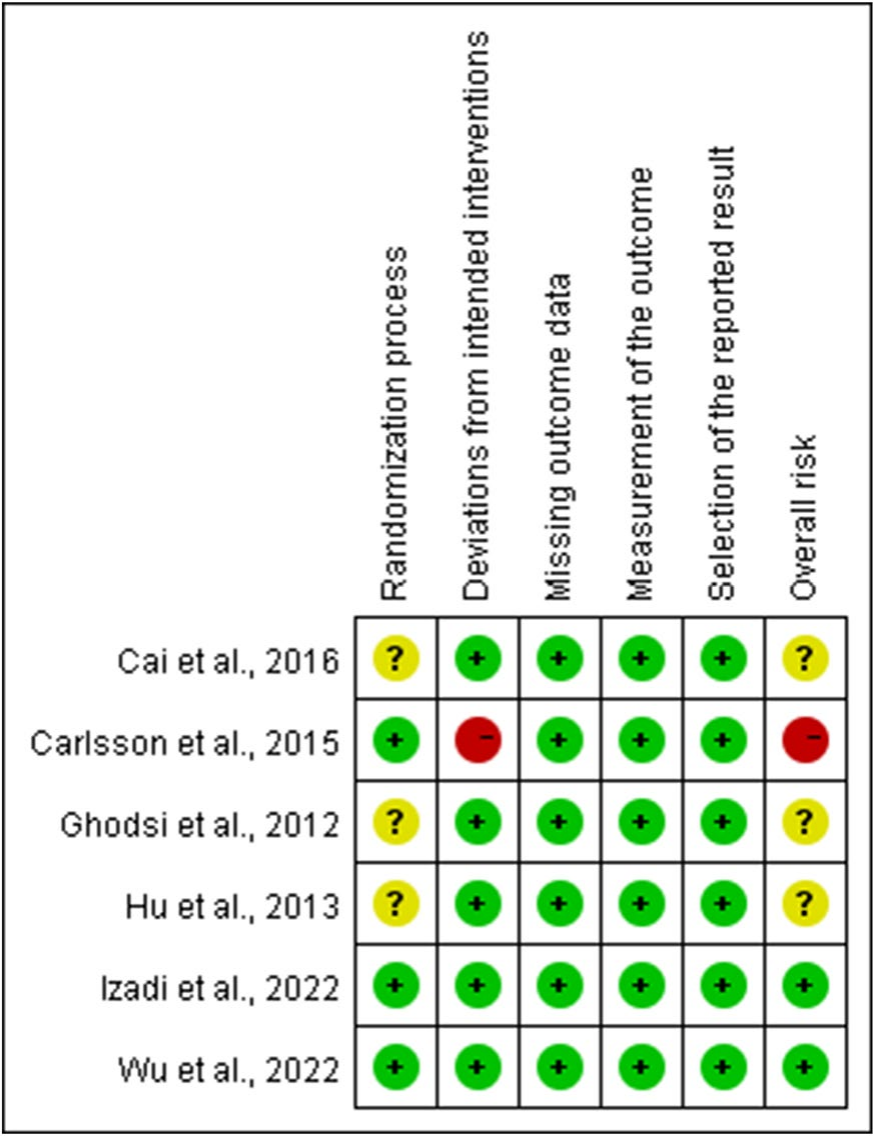
Risk of bias summary for randomized controlled trials

For RCTs, one trial was rated as "high risk", three as "some concerns", and two as "low risk". Domains of bias due to the randomization process and deviations from intended interventions were major methodological concerns. Figures 2 and 3 depict the risk of bias for RCTs. Regarding nRCTs, there was an overall serious risk of bias in three studies, a moderate risk of bias in four, and a low risk of bias in one. All studies except one showed moderate to severe risk of bias due to potential confounding factors, deviations from intended interventions, and missing data due to missing patients. Table 2 presents the risk of bias for nRCTs. In summary, although two RCTs and one nRCT demonstrated a low risk of bias, most of the studies showed varying levels of concern about bias, which may well have affected the credibility of their findings.

**Table 2:** Risk of bias assessment for non-randomized controlled trials

Study	Bias due to confounding	Bias in selection of participants into the study	Bias in classification of interventions	Bias due to deviations from intended interventions	Bias due to missing data	Bias in measurement of outcomes	Bias in selection of the reported result	Overall bias
Lu et al. (2021)	Low	Low	Low	Low	Serious	Low	Low	Serious
Yu et al. (2011)	Serious	Low	Low	Low	Low	Low	Low	Serious
Gu et al. (2014)	Serious	Low	Low	Moderate	Low	Low	Low	Serious
Gu et al. (2017)	Low	Low	Low	Low	Moderate	Low	Low	Moderate
Walicka et al. (2018)	Moderate	Low	Low	Low	Low	Low	Low	Moderate
Ye et al. (2017)	Moderate	Low	Low	Low	Low	Low	Low	Moderate
Giannopoulou et al. (2014)	Moderate	Low	Low	Low	Low	Low	Low	Moderate
Zhao et al. (2012)	Low	Low	Low	Low	Low	Low	Low	Low

### Study outcomes

The intervention effects of these trials are presented in Table 3 and Table 4. HbA1c and C peptide were assessed in all studies, insulin requirements in 11, FBG in 8, and PBG in 3. In ten studies, improvements in at least one of the outcomes of interest after MSCs intervention were significant compared to controls or baseline. In the other four studies, all outcomes had not significantly improved in comparison with the control group.

**Table 3:** Results of study outcomes

Study	Results
Carlsson et al. (2015)	Compared to the control group, the SC group ↑: Peak C-peptide (P < 0.05), AUC C-peptide (P < 0.05) No effect: HbA1c, insulin requirements, fasting C-peptide
Izadi et al. (2022)	Compared to the control group, the SC group ↓: HbA1c (P = 0.043) No effect: FBG, PBG, insulin requirements, C-peptide
Hu et al. (2013)	Compared to the control group, the SC group ↑: Fasting C-peptide, C-peptide/glucose ratio ↓: HbA1c (P < 0.05), PBG (P < 0.05), insulin requirements (P < 0.001) No effect: FBG
Lu et al. (2021)	Compared to the baseline, the SC group No effect: HbA1c, insulin requirements, fasting C-peptide, postprandial C-peptide Compared to the baseline, the control group No effect: HbA1c, insulin requirements, fasting C-peptide, postprandial C-peptide
Yu et al. (2011)	Compared to the baseline, the SC group ↑: 2 h postprandial C-peptide (P < 0.01) ↓: HbA1c ( P < 0.01), FBG (P < 0.05) No effect: PBG, insulin requirements, fasting C-peptide, 1 h postprandial C-peptide Compared to the baseline, the control group ↓: 1 h postprandial C-peptide (P < 0.05), 2 h postprandial C-peptide (P < 0.01) No effect: HbA1c, FBG, PBG, insulin requirements, fasting C-peptide
Ghodsi et al. (2012)	Compared to the control group, the SC group No effect: HbA1c, FBG, fasting C-peptide
Gu et al. (2014)	Compared to the control group, the SC group ↑: HbA1c (P < 0.05) No effect: Insulin requirements, C-peptide
Gu et al. (2017)	Compared to the control group, the SC group ↑: Peak C-peptide (P < 0.001), AUC C-peptide (P < 0.001) ↓: Insulin requirements (P < 0.01) No effect: HbA1c
Walicka et al. (2018)	Compared to the control group, the insulin-treated SC group ↑: Fasting C-peptide (P < 0.05), postprandial C-peptide (P < 0.005) No effect: HbA1c, FBG Compared to the control group, the insulin-free SC group ↑: Postprandial C-peptide (P < 0.05) No effect: HbA1c, FBG, fasting C-peptide
Ye et al. (2017)	Compared to the control group, the SC group ↑: Fasting C-peptide (P = 0.031), AUC C-peptide (P = 0.002) ↓: Insulin requirements (P = 0.004) No effect: HbA1c, FBG
Giannopoulou et al. (2014)	Compared to the control group, the SC group No effect: HbA1c, insulin requirements, peak C-peptide, AUC C-peptide
Zhao et al. (2012)	Compared to the baseline, the SC group having moderate T1D with some residual b cell function (Group A) ↑: Fasting C-peptide (P < 0.05), postprandial C-peptide (P < 0.05) ↓: HbA1c (P < 0.05), insulin requirements (P < 0.05) Compared to the baseline, the SC group having severe T1D with no residual pancreatic islet b cell function (Group B) ↑: Fasting C-peptide (P < 0.05), postprandial C-peptide (P < 0.05) ↓: HbA1c (P < 0.05), insulin requirements (P < 0.05) Compared to the baseline, the control group No effect: HbA1c, insulin requirements, fasting C-peptide, postprandial C-peptide
Cai et al. (2016)	Compared to the control group, the SC group ↑: Fasting C-peptide (P < 0.01), AUC C-peptide (P = 0.013), AUC insulin (P = 0.027) ↓: HbA1c (P < 0.01), FBG (P < 0.01), insulin requirements (P < 0.01)
Wu et al. (2022)	Compared to the control group, the SC group ↑: Fasting C-peptide (P < 0.001) ↓: HbA1c (P = 0.029) No effect: FBG, insulin requirements

**Table 4:** Effects of different types of cells on glycemic outcomes

Type of cells	Included studies	Improved outcomes
BM-MSCs	Carlsson et al. (2015)	Peak C-peptide, AUC C-peptide
	Izadi et al. (2022)	HbA1c
UC-MSCs	Hu et al. (2013)	Fasting C-peptide, C-peptide/glucose ratio, HbA1c, PBG, insulin requirements
	Lu et al. (2021)	None
	Yu et al. (2011)	2 h postprandial C-peptide HbA1c, FBG
AHSCT	Ghodsi et al. (2012)	None
	Gu et al. (2014)	None
	Gu et al. (2017)	Peak C-peptide, AUC C-peptide, insulin requirements
	Walicka et al. (2018)	Fasting C-peptide, postprandial C-peptide
	Ye et al. (2017)	Fasting C-peptide, AUC C-peptide, insulin requirements
CB-SCs	Giannopoulou et al. (2014)	None
	Zhao et al. (2012)	Fasting C-peptide, postprandial C-peptide, HbA1c, insulin requirements
UC-MSCs + aBM-MNCs	Cai et al. (2016)	Fasting C-peptide AUC C-peptide, AUC insulin, HbA1c, FBG, insulin requirements
	Wu et al. (2022)	Fasting C-peptide, HbA1c

#### Effect of BM-MSCs

Two studies reported the benefit of BM-MSCs in newly-diagnosed T1D. In an open, single-center RCT conducted by Carlsson et al. (2015), HbA1c, insulin requirements, and fasting C-peptide did not differ significantly between patients administered with MSCs and those only receiving insulin therapy. Both C-peptide peak values and area under curve for C-peptide (AUC C-peptide) were preserved and even increased in patients treated with MSCs, but these responses were reduced in the control group, indicating a statistical difference between the two groups. In a triple-blinded parallel RCT performed by Izadi et al. (2022), the effect was measured by change of outcomes from baseline at 3, 6, 9 and 12 months. There was no distinct difference in terms of FBG, PBG, insulin dose and C-peptide between the intervention and the control arms. MSCs significantly reduced HbA1c at 12 months (P = 0.043), but the change was insignificant in other time points.

#### Effect of UC-MSCs

Three studies involved UC-MSCs therapy for T1D. Hu et al. (2013) reported that in a double-blind RCT, PBG levels in the MSCs group fell to their lowest level at 1 year after transplantation, and the difference was distinct when compared to placebo. A progressive decrease was seen in HbA1c with a lowest level at 6-month follow-up in the treatment group (6 months 5.5 ± 0.67%, baseline 6.8 ± 0.57%, respectively), which remained lower than that in the placebo group. Fasting C-peptide rose to its highest level at 1 year in the MSCs group, but gradually declined in controls. By comparison of the MSCs group and control group, both fasting C-peptide and C-peptide/glucose ratio values were statistically different at the end of follow-up. Daily insulin dosage of MSCs-treated patients was gradually reduced, whereas it was increased with placebo, which exhibited remarkable variation between groups. As for FBG, no statistically significant change was found. Yu et al. (2011) set up a non-randomized concurrent controlled trial that showed the positive effect of UC-MSCs transplantation on newly-onset T1D. Changes in FBG, PBG, HbA1c, C-peptide and insulin dose were measured before and after receiving transplantation. At 9 months follow-up, 2-h postprandial C-peptide and FBG increased significantly after MSCs intervention, however, both 1-h and 2-h postprandial C-peptide levels decreased in the control group. HbA1c decreased from 10.53 ± 0.98% to 6.57 ± 0.78% in the MSCs group, without significantly changes in the control group. Relative to the baseline, the remaining metrics remained constant within the group. In the trial carried out by Lu et al. (2021), there were no significant discrepancies in HbA1c, insulin doses, fasting and postprandial C-peptide within both groups compared to the baseline.

#### Effect of AHSCT

Five studies were designed to explore the effects of AHSCT on T1D. In one study assessing the clinical effect of AHSCT in treating children (ranged from 1.5 to 12.5 years old) who were newly-diagnosed T1D (Gu et al. 2014), the end of follow-up time was 3 to 5 years. Whether during the short-term follow-up (10.7 ± 4.2 months after AHSCT) or the long-term follow-up (4.2 ± 1.8 years after AHSCT), the control group remained below the AHSCT intervention group on HbA1c (P < 0.05). However, serum C-peptide and insulin dosages did not significantly differ between groups. A phase-2 prospective, parallel non-randomized trial aimed at evaluating the clinical benefits of AHSCT in adolescents with newly-diagnosed T1D (Gu et al. 2017). During 48-month follow-up, HbA1c level dropped dramatically 3 months post treatment from baseline in both the AHSCT and the control groups, but no statistical difference between groups was discovered over time. The daily dose of insulin was sharply declined in AHSCT participants, with a minimum at 3-month follow-up, whereas it showed no significant change over the follow-up duration. The dose of insulin was significantly different between the two groups, except at 18 months, where the AHSCT patients had lower levels. C-peptide peak value in the AHSCT group reached its highest level 6 months after transplantation. In the insulin arm, it reached the highest at 3 months. Compared to the baseline, in the AHSCT group, AUC C-peptide reached its peak at 6 months, and in the insulin arm, it was the highest at 3 months. Peak C-peptide and AUC C-peptide showed significant differences between the two groups after 3 months and until the end of therapy, which were higher in AHSCT subjects. Walicka et al. (2018) performed a non-randomized, interventional study in which patients were administered with autologous peripheral blood stem cell transplantation (APBSCT) as the intervention group or received insulin therapy as a control. The APBSCT subjects were classified into: those who had not received insulin until the 48th month follow-up time (the insulin-treated group), and those who took it at any time over the 48th month follow-up time (the insulin-free group). All groups had an improvement in HbA1c at 24 and 36 months, with the level remarkably lower in the insulin-free group. At 48th month, HbA1c in the insulin-free group had a slight increase, while in other groups HbA1c decreased. After 12 months, FBG remained low in the transplanted patients, while it began to increase in the control group. It significantly lowered in both APBSCT groups in contrast to the control group up to month 36. Fasting and postprandial C-peptide in both APBSCT groups were at least significantly greater than the control group at certain period. Ye et al. (2017) found that 12 months after transplantation, HbA1c was remarkably decreased in the insulin-only group compared to the baseline, while FBG, fasting C-peptide, AUC C-peptide, and insulin requirements were not statistically different. In comparison with the control group, AHSCT subjects achieved remarkably higher fasting C-peptide at 12 months and AUC C-peptide, with declined insulin requirements. In a double-blind, placebo controlled trial, Ghodsi et al. (2012) concluded that fetal liver-derived hematopoietic stem cell transplantation played no remarkable role in HbA1c, FBG, and serum C-peptide.

#### Effect of CB-SCs

Two studies investigated the efficacy of CB-SCs for T1D. In a clinical trial, Zhao et al. (2012) enrolled 15 T1D to investigate the effectiveness of Stem Cell Educator. In Group A, SCs-treated participants had moderate T1D with residual beta-cell function, while those in Group B had severe T1D without beta-cell function. Patients in group A showed an improvement in fasting C-peptide at 12 and 24 weeks, and Group B had continuous improvement at each follow-up. Postprandial C-peptide in Group A improved at 4 and 12 weeks, while Group B demonstrated remarkable improvement at 12 weeks and up to 40-week. C-peptide did not exhibit significant changes in the control group. Daily insulin dosages were reduced 38% in Group A at 12 weeks compared to the baseline (22 ± 1.8 vs. 36 ± 13.2 units/day) and 25% in Group B (36 ± 4.4 vs. 48 ± 7.4 units/day), while controls demonstrated no differences. Decreases were sustained in groups A and B during 24 weeks of follow-up. HbA1c in Group A was significantly lowered at 4 weeks compared to baseline data (7.67 ± 1.03% vs. 8.73 ± 2.49%). After 12 weeks of treatment, there was a decrease of 1.68 ± 0.42% in group B, but not in controls. Giannopoulou et al. (2014) demonstrated that 6th- and 12th-month HbA1c, insulin requirements, AUC C-peptide and peak C-peptide levels had no statistical discrepancy between groups. Median daily insulin requirements in the infusion group increased by 36.0% after 12 months compared to baseline. The controls showed increased 6th- and 12th-month insulin requirements compared to baseline, but levels did not significantly differ from that in the infused patients.

#### Effect of UC-MSCs plus aBM-MNCs

Two studies aimed to explore the benefits of UC-MSCs plus aBM-MNCs in established T1D without immunotherapy. Cai et al. (2016) reported a pilot open-label RCT with metabolic parameters improved in stem cell transplantation (SCT) participants at 1 year. AUC C-peptide in SCT recipients was raised by 105.7%, while that in the control group decreased by 7.7%. HbA1c decreased in the SCT group from 8.6 ± 0.81% to 7.5 ± 1.0%, while in the control group it increased from 8.7 ± 0.9% to 8.8 ± 0.9%. FBG was reduced by 24.4% in the SCT group, and 4.3% in the control group at 12 months. In SCT subjects, there was a striking reduction in insulin demand of 29.2%, while no change was seen in controls. Fasting C-peptide levels were constant in the control group, but they markedly increased at 9th and 12th months of SCT. The results showed that AUC C-peptide, HbA1c, FBG, insulin demand, and fasting C peptide had a significant amelioration with SCT. Wu et al. (2022) focused on the long-term impact following SCT with 8 years of follow-up. In the SCT group, HbA1c lowered from the baseline of 8.57 ± 0.97% to 7.89 ± 1.28% at 8 years, while it kept steady in the control group. HbA1c between groups reached statistical different at 1 and 8 years. Similarly, in the SCT group, FBG reduced from 200.06 ± 59.26 mg/dL to 168.69 ± 22.70 mg/dL at 8 years, whereas it remained steady in the control group. FBG had a remarked decrease in the SCT group at 1 year, but there was no statistically significant difference between groups at 8 years. Insulin doses in the SCT group were reduced from the baseline at 1 year (0.66 ± 0.16 IU/d/kg vs. 0.90 ± 0.24 IU/d/kg), which were remarkably lower than that in the control group. However, the differences between groups demonstrated no significance at 8 years. Fasting C-peptide in the SCT group was 0.064 ± 0.031 pmol/mL at 1 year and 0.048 ± 0.021 pmol/mL at 8 years, which had a significant higher value than that in the control group.

## Discussion

The objective of the current systematic review was to assess the impact of MSCs on T1D. Despite heterogeneity in the studies, 10 out of 14 studies demonstrated a significant reduction in at least one glycemic outcome or an improved response compared to control groups (Cai et al. 2016; Carlsson et al. 2015; Gu et al. 2017; Hu et al. 2013; Izadi et al. 2022; Walicka et al. 2018; Wu et al. 2022; Ye et al. 2017; Yu et al. 2011; Zhao et al. 2012). Considering that T1D is a global public health concern associated with substantial disability and reduced lifespan, these findings are meaningful. Most studies showed noteworthy decreases in FBG, PBG, HbA1c, insulin requirement as well as increases in C-peptide levels following SCT. Interestingly, four studies did not observe any effect of MSCs on glycemic outcomes (Ghodsi et al. 2012; Giannopoulou et al. 2014; Gu et al. 2014; Lu et al. 2021). These conflicting results may be attributed to flaws in study design. For instance, Lu et al. (2021) had a major imbalance in baseline insulin requirement between groups and included participants with a wide age range (8–55 years old). Ghodsi et al. [14] (2012) involved individuals with varying ages (ranging from 10 to 58 years old) and durations of diabetes (ranging from 6 months to 11 years). Giannopoulou et al. (2014) had significantly different baseline ages between the two groups which could have contributed to the negative results. Additionally, the short follow-up period (1 year) in these studies might explain why there were no significant changes observed in glycemic outcomes. Gu et al. (2014) discovered that the patients and their parents in the insulin group took part in the educational seminar for diabetic children, and strictly controlled the frequency of glucose monitoring, recommended dose, diet and exercise, which could account for their better HbA1c in this group.

Despite heterogeneity among the studies reviewed, our findings support an association between MSCs therapy and better glycemic control for T1D patients. This aligns with a recently published meta-analysis (Wu et al. 2020), which reported a significant effect on reducing HbA1c levels and improving C-peptide levels among T1D patients after one year follow-up of SCT.

Various strategies such as adopting a healthy diet, using hypoglycemic agents, and administering insulin have been implemented for diabetes management (Holt et al. 2021), but most conventional medicines have some undesirable side effects and this often contributes to diabetic treatment failure (Tan et al. 2019). Thus, stem cell replacement therapy holds great potential as a novel approach for treating T1D, with MSCs infusion being the most extensively studied method in clinical trials. Numerous successful cases have demonstrated the renewal and differentiation of stem cells (SCs) into insulin-producing cells (IPCs), which plays a key role in their therapeutic effect on T1D. For instance, Neshati et al. [31] (2010) discovered that by treating cells with high glucose concentration, beta-mercaptoethanol, and nicotinamide, functional IPCs were differentiated and able to reverse hyperglycaemia in a diabetic rat model. Sarang and Viswanathan (2016) successfully differentiated UC-MSCs into IPCs expressing multiple markers associated with pancreatic islet cell development and function; these IPCs were capable of releasing glucose-induced insulin in a dose-dependent fashion. It is known that T1D is a complex condition influenced by genetic, epigenetic, and environmental factors that impact adaptive and innate effector cell populations leading to chronic inflammation within pancreatic islets (Clark et al. 2017). The activation of antigen-presenting cells due to insulitis triggers CD4 + helper T cell activation leading to cytokine release. Cytokines then activate CD8 + cytotoxic-T cells, thus resulting in β-cell destruction (Tomita 2017). MSCs participate in the regulation of peripheral immune response via secreting anti-inflammatory and immunomodulatory factors, including transformed growth factor-β (TGF-β), interleukin-4 (IL-4), interleukin-10 (IL-10) and others. Besides, they decrease the production of proinflammatory factors, such as interferon-γ (IFN-γ), tumor necrosis factor-α (TNF-α), interleukin-6 (IL-6), interleukin-17 (IL-17), and interleukin-12 (IL-12) (Brini et al. 2017). As a result, MSCs decrease the frequency of CD4 + and CD8 + T cells, and enhance the quantity of regulatory T cells (Treg). Moreover, in vivo studies have shown that MSCs induce macrophage polarization from pro-inflammatory M1 phenotype to anti-inflammatory M2 phenotype through the secretion of prostaglandin E2 (PGE2) and IL-10 in response to PGE2 (Liu et al. 2020). MSCs promote tissue regeneration by paracrine mechanism, secreting factors like vascular endothelial growth factor (VEGF), epithelial growth factor (EGF), hepatocyte growth factor (HGF), and platelet-derived growth factor (PDGF) (Dabrowski et al. 2017), which reduce islet cell apoptosis and improve islet survival.

MSCs could delay the onset of experimental autoimmune diabetes (EAD) in immunized RIP-B7.1 mice through anti-inflammatory and immunomodulatory responses (Lachaud et al. 2023). In clinical, the United States Food and Drug Administration (FDA) approved Tzield (teplizumab-mzwv), an anti-CD3-directed antibody, as the first immunomodulatory treatment to delay the onset of stage 3 T1D in adults and pediatric patients aged 8 years and older with stage 2 T1D (Evans-Molina and Oram 2023). MSCs and immunomodulatory therapies that delay or prevent T1D currently encounter similar obstacles with a small percent of eligible participants enrolling into trials. Although autoimmune biomarkers and antibodies are examined to detect the disease progression and benefit of treatment, for people who are in the presymptomatic stages of T1D, screening remains still a main tissue (Kinney et al. 2023). Besides, challenges in cost-effectiveness, adverse events, and accessibility of treatment are urgent to deal with.

Few studies have reported undesirable adverse effects from MSCs, however, there still are some concerns about severe adverse events including tumorigenicity and microvascular thrombosis (Gleeson et al. 2015; Guillamat-Prats 2022). According to Walicka et al. (2018), one out of twenty-four patients who underwent AHSCT with high-dose cyclophosphamide plus anti-thymocyte globulin in the course of neutropenia died of Pseudomonas sepsis. While immune function was restored following AHSCT, conditioning with immunoablation rendered patients susceptible to opportunistic infections. Therefore, there is still a need for standardization and improvement of procedures to ensure the safety of MSCs in clinical practice.

The present systematic review represents the latest evaluation on whether MSCs contribute significantly to improved glycemic control in T1D. The strength of this review lies in its rigorous methodology, which includes explicit eligibility criteria and an extensive search across comprehensive databases. The literature retrieval employed thorough strategies to encompass all relevant aspects associated with the aims of this review. However, heterogeneity among the included studies, particularly regarding stem cell types, duration of diabetes, and follow-up periods limits this systematic review. Furthermore, due to heterogeneity in outcome measures, comparators, and methodologies used across studies, conducting a meta-analysis is not feasible. It should also be noted that double-blind studies are not always possible in MSCs research, leading to potential bias in the included studies. Future investigations should focus on examining both short-term and long-term impact of various kinds of MSCs on glycemic measures. Larger sample sizes and longer follow-up periods are issues to be addressed for future research endeavors. The utilization of pre-defined and standardized outcome measures will facilitate determining the effectiveness of MSCs for T1D while enabling comparisons between study findings.

This systematic review highlights the positive impacts observed from utilizing MSCs in individuals with T1D. The potential therapeutic value of MSCs remains promising; however, further investigation is required due to variations in sample sizes and concerns regarding study quality. To get a clearer picture of the underlying mechanisms and establish whether MSCs are really beneficial to T1D, additional experimental and clinical trials with comprehensive baseline assessments and extended follow-up are necessary.

### Supplementary Information

Below is the link to the electronic supplementary material.

Supplementary file 1 (PDF 20 KB)
