# Supplementary material for: Efficacy of mesenchymal stromal cells in the treatment of type 1 diabetes: a systematic review
Source: Cell Tissue Bank. 2024 Feb 21;25(2):663–76. doi: 10.1007/s10561-024-10128-1 (PMC11143029; doi:10.1007/s10561-024-10128-1)
Supplement: Supplementary file 1 — (PDF 20 KB) [file 10561_2024_10128_MOESM1_ESM.pdf]

Search strategy

Web of Science

#1 TS=("Diabetes mellitus, Type 1" OR "Diabetes Mellitus, Insulin-Dependent" OR "Diabetes Mellitus, Insulin Dependent" OR "Insulin-Dependent Diabetes Mellitus" OR "Diabetes Mellitus, Juvenile-Onset" OR "Diabetes Mellitus, Juvenile Onset" OR "Juvenile-Onset Diabetes Mellitus" OR "IDDM" OR "Juvenile-Onset Diabetes" OR "Diabetes, Juvenile-Onset" OR "Juvenile Onset Diabetes" OR "Diabetes Mellitus, Sudden-Onset" OR "Diabetes Mellitus, Sudden Onset" OR "Sudden-Onset Diabetes Mellitus" OR "Type 1 Diabetes Mellitus" OR "Insulin-Dependent Diabetes Mellitus 1" OR "Insulin Dependent Diabetes Mellitus 1" OR "Type 1 Diabetes" OR "Diabetes, Type 1" OR "Diabetes Mellitus, Type I" OR "Diabetes Mellitus, Brittle" OR "Brittle Diabetes Mellitus" OR "Ketosis-Prone Diabetes Mellitus" OR "Diabetes, Autoimmune" OR "Autoimmune Diabetes")

#2 TS=("Stem Cells" OR "Cell, Stem" OR "Cells, Stem" OR "Stem Cell" OR "Progenitor Cells" OR "Cell, Progenitor" OR "Cells, Progenitor" OR "Progenitor Cell" OR "Mother Cells" OR "Cell, Mother" OR "Cells, Mother" OR "Mother Cell" OR "Colony-Forming Unit" OR "Colony Forming Unit" OR "Colony-Forming Units" OR "Colony Forming Units")

#3 #1 and #2

PubMed

#1 "Diabetes mellitus, Type 1"[MeSH Terms]

#2 "Diabetes mellitus, Type 1"[Title/Abstract] OR "Diabetes Mellitus, Insulin-Dependent"[Title/Abstract] OR "Diabetes Mellitus, Insulin Dependent"[Title/Abstract] OR "Insulin-Dependent Diabetes Mellitus"[Title/Abstract] OR "Diabetes Mellitus, Juvenile-Onset"[Title/Abstract] OR "Diabetes Mellitus, Juvenile Onset"[Title/Abstract] OR "Juvenile-Onset Diabetes Mellitus"[Title/Abstract] OR "IDDM"[Title/Abstract] OR "Juvenile-Onset Diabetes"[Title/Abstract] OR "Diabetes, Juvenile-Onset"[Title/Abstract] OR "Juvenile Onset Diabetes"[Title/Abstract] OR "Diabetes Mellitus, Sudden-Onset"[Title/Abstract] OR "Diabetes Mellitus, Sudden Onset"[Title/Abstract] OR "Sudden-Onset Diabetes Mellitus"[Title/Abstract] OR "Type 1 Diabetes Mellitus"[Title/Abstract] OR "Insulin-Dependent Diabetes Mellitus 1"[Title/Abstract] OR "Insulin Dependent Diabetes Mellitus 1"[Title/Abstract] OR "Type 1 Diabetes"[Title/Abstract] OR "Diabetes, Type 1"[Title/Abstract] OR "Diabetes Mellitus, Type I"[Title/Abstract] OR "Diabetes Mellitus, Brittle"[Title/Abstract] OR "Brittle Diabetes Mellitus"[Title/Abstract] OR "Ketosis-Prone Diabetes Mellitus"[Title/Abstract] OR "Diabetes, Autoimmune"[Title/Abstract] OR "Autoimmune Diabetes"[Title/Abstract]

#3 #1 or #2

#4 "Stem Cells"[MeSH Terms]

#5 "Stem Cells"[Title/Abstract] OR "Cell, Stem"[Title/Abstract] OR "Cells, Stem"[Title/Abstract] OR "Stem Cell"[Title/Abstract] OR "Progenitor Cells"[Title/Abstract] OR "Cell, Progenitor"[Title/Abstract] OR "Cells, Progenitor"[Title/Abstract] OR "Progenitor Cell"[Title/Abstract] OR "Mother Cells"[Title/Abstract] OR "Cell, Mother"[Title/Abstract] OR "Cells, Mother"[Title/Abstract] OR "Mother Cell"[Title/Abstract] OR "Colony-Forming Unit"[Title/Abstract] OR "Colony Forming Unit"[Title/Abstract] OR "Colony-Forming Units"[Title/Abstract] OR "Colony Forming Units"[Title/Abstract]

#6 #4 or #5

#7 #3 and #6

Embase

#1 'insulin dependent diabetes mellitus'/exp

#2 'brittle diabetes':ti,ab,kw OR 'brittle diabetes mellitus':ti,ab,kw OR 'diabetes mellitus type 1':ti,ab,kw OR 'diabetes mellitus type i':ti,ab,kw OR 'diabetes mellitus, brittle':ti,ab,kw OR 'diabetes mellitus, insulin dependent':ti,ab,kw OR 'diabetes mellitus, insulin-dependent':ti,ab,kw OR 'diabetes mellitus, juvenile onset':ti,ab,kw OR 'diabetes mellitus, type 1':ti,ab,kw OR 'diabetes mellitus, type i':ti,ab,kw OR 'diabetes type 1':ti,ab,kw OR 'diabetes type i':ti,ab,kw OR 'diabetes, juvenile':ti,ab,kw OR 'dm 1':ti,ab,kw OR 'early onset diabetes mellitus':ti,ab,kw OR 'iddm':ti,ab,kw OR 'insulin dependent diabetes':ti,ab,kw OR 'insulin-dependent diabetes mellitus':ti,ab,kw OR 'juvenile diabetes':ti,ab,kw OR 'juvenile diabetes mellitus':ti,ab,kw OR 'juvenile onset diabetes':ti,ab,kw OR 'juvenile onset diabetes mellitus':ti,ab,kw OR 'ketoacidotic diabetes':ti,ab,kw OR 'labile diabetes mellitus':ti,ab,kw OR 'mckusick 22210':ti,ab,kw OR 't1dm':ti,ab,kw OR 'type 1 diabetes':ti,ab,kw OR 'type 1 diabetes mellitus':ti,ab,kw OR 'type i diabetes':ti,ab,kw OR 'type i diabetes mellitus':ti,ab,kw OR 'insulin dependent diabetes mellitus':ti,ab,kw

#3 #1 or #2

#4 'stem cell'/exp

#5 'cell, stem':ti,ab,kw OR 'precursor cell':ti,ab,kw OR 'progenitor cell':ti,ab,kw OR 'stem cells':ti,ab,kw OR 'stem cell':ti,ab,kw

#6 #4 or #5

#7 #3 and #6

Cochrane Library

#1 MeSH descriptor: [Diabetes Mellitus, Type 1] explode all trees

#2 ("Diabetes Mellitus, Type 1" OR "Insulin-Dependent Diabetes Mellitus" OR "Diabetes, Juvenile-Onset" OR "Diabetes Mellitus, Insulin-Dependent, 1" OR "Diabetes Mellitus, Sudden Onset" OR "IDDM" OR "Diabetes, Type 1" OR "Diabetes Mellitus, Type I" OR "Type 1 Diabetes" OR "Diabetes Mellitus, Insulin Dependent" OR "Insulin-Dependent Diabetes Mellitus 1" OR "Insulin Dependent Diabetes Mellitus 1" OR "Sudden-Onset Diabetes Mellitus" OR "Juvenile-Onset Diabetes" OR "Type 1 Diabetes Mellitus" OR "Juvenile Onset Diabetes" OR "Juvenile-Onset Diabetes Mellitus" OR "Diabetes Mellitus, Juvenile Onset" OR "Diabetes Mellitus, Juvenile-Onset" OR "Diabetes Mellitus, Sudden-Onset" OR "Diabetes Mellitus, Insulin-Dependent" OR "Autoimmune Diabetes" OR "Diabetes, Autoimmune" OR "Diabetes Mellitus, Brittle" OR "Brittle Diabetes Mellitus" OR "Diabetes Mellitus, Ketosis-Prone" OR "Diabetes Mellitus, Ketosis Prone" OR "Ketosis-Prone Diabetes Mellitus"):ti,ab,kw

#3 #1 or #2

#4 MeSH descriptor: ["Stem Cells"] explode all trees

#5 ("Stem Cell" OR "Cell, Stem" OR "Cells, Stem” OR "Mother Cells" OR "Mother Cell" OR "Cells, Progenitor" OR "Cells, Mother" OR "Progenitor Cell" OR "Cell, Progenitor" OR "Cell, Mother" OR "Progenitor Cells" OR "Colony Forming Units" OR "Colony Forming Unit" OR "Colony-Forming Units" OR "Colony-Forming Unit"):ti,ab,kw

#6 #4 or #5

#7 #3 and #6
